# Complementary and Alternative Medicine in the Management of Perimenopausal Sleep Disturbances: A Scoping Review

**DOI:** 10.7759/cureus.114565

**Published:** 2026-08-15

**Authors:** Anisa N Ramcharitar-Bourne, Karthik Madhavan, Mohammed M Mohiuddin, Akanksha Mehra

**Affiliations:** 1 Rehabilitation Sciences and Wellness, University of New Haven, West Haven, USA; 2 Population Health and Leadership, University of New Haven, West Haven, USA; 3 Department of Cardiac Imaging, Houston Methodist Hospital, Houston, USA

**Keywords:** botanical remedies, cam, complementary medicine, insomnia, integrative medicine, mind-body therapies, perimenopause, scoping review, sleep disturbance, women’s health

## Abstract

Perimenopause is a natural midlife transition characterized by hormonal and neurological changes that disrupt thermoregulation and circadian sleep-wake regulation, thereby contributing to sleep disturbances. These disturbances are associated with poorer mental health, increased cardiometabolic risk, reduced work productivity, and lower quality of life (QoL). Treatment options include hormonal and nonhormonal therapies, with complementary and alternative medicine (CAM) representing a commonly used nonhormonal approach. However, the evidence supporting CAM for perimenopausal sleep disturbances remains unclear. This scoping review mapped the available literature on CAM interventions for sleep disturbances in healthy perimenopausal women.

The Joanna Briggs Institute (JBI) methodology and Preferred Reporting Items for Systematic Reviews and Meta-Analyses Extension for Scoping Reviews (PRISMA-ScR) guidelines were followed. Comprehensive searches were performed in PubMed, CINAHL, Scopus, Cochrane Library, and Web of Science for English-language studies published between 2014 and 2024. Four reviewers independently screened records and full texts using Covidence, with disagreements resolved by consensus. Data from eligible studies were charted and synthesized descriptively by CAM modality, study design, and the direction of sleep-related outcomes.

Thirty-two studies, primarily randomized controlled trials (RCTs), were included, with most reporting improvements in sleep quality. However, substantial heterogeneity in interventions, outcome measures, and study quality limits the strength of recommendations. Larger, standardized trials with objective sleep assessments, more rigorous study designs, greater population diversity, and longer follow-up periods are needed.

## Introduction and background

Menopause marks a major physiological transition in women’s lives, defined by the permanent cessation of menstruation following the depletion of ovarian follicles and a decline in estrogen production. Perimenopause, as described in the Stages of Reproductive Aging Workshop+10 (STRAW+10) framework, represents the transition period leading up to menopause and is characterized by hormonal fluctuations, irregular menstrual cycles, and the onset of early symptoms [1,2]. Clinically, perimenopause begins with the first noticeable menstrual cycle irregularity and extends until one year after the final menstrual period, encompassing both early and late transition stages [3-5].

Perimenopausal hormonal shifts can lead to vasomotor disturbances [6-8], sleep difficulties [9,10], cognitive changes, mood fluctuations, migraines, osteoporosis, and adverse metabolic and cardiovascular changes [5,10-13], although symptom experiences vary widely across cultures and populations. Beyond its biological dimensions, perimenopause represents a significant yet often underrecognized public health issue. Evidence shows substantial heterogeneity in symptom prevalence and interpretation, shaped by ethnicity [14], social norms, stigma, and access to information and care [1]. Qualitative work also highlights that women’s lived experiences are often insufficiently addressed within healthcare systems, despite the potential for this transition to affect daily functioning, identity, and well-being [2,15].

Sleep disturbance is among the most common concerns during the perimenopausal transition. The Study of Women’s Health Across the Nation (SWAN) reports sleep disturbances in up to 47% of perimenopausal women, with insomnia being the most common manifestation. During perimenopause, declining estrogen and progesterone levels can alter neurotransmitter activity, thermoregulation, and arousal thresholds, contributing to insomnia, nighttime awakenings, and reduced sleep efficiency [9,16,17]. These disturbances are associated with adverse health outcomes and diminished quality of life (QoL) [17,18]. Although hormone replacement therapy (HRT) may positively affect sleep, particularly when sleep disruption is associated with vasomotor symptoms, some women cannot or prefer not to use HRT and may seek complementary and alternative medicine (CAM) or other non-pharmacological approaches [19-21].

CAM encompasses a heterogeneous group of practices that may be used alongside or in place of conventional care. Commonly described categories include whole medical systems (e.g., Ayurveda and Traditional Chinese Medicine (TCM)), mind-body therapies (e.g., yoga, muscle relaxation, mindfulness-based stress reduction (MBSR), meditation, tai chi), biologically based practices (e.g., botanicals and dietary supplements), energy healing (e.g., reiki), and manipulative or body-based therapies (e.g., massage, chiropractic therapy, and reflexology) [22,23]. CAM use is common among midlife women, and national data indicate that approximately 46% of American women aged 40-59 years use CAM, with estimates of 52-76% reported in studies from the United States, Canada, and Australia [24].

Women’s motivations for using CAM include alignment with personal beliefs, perceived holistic benefits, and clinician recommendations [25]. CAM use among perimenopausal women has also been reported in the context of anxiety, somatic symptoms, and vasomotor symptoms [26], which may in turn contribute to improved overall sleep quality. Approaches such as relaxation therapies, dietary supplements, herbal remedies, yoga, meditation, acupuncture, and music therapy have been used to manage menopausal symptoms, including those affecting sleep [26,27]. The prevalence and diversity of CAM use among midlife women underscore the importance of understanding the evidence supporting these approaches for sleep outcomes specifically during perimenopause.

Given the breadth and heterogeneity of the literature, including variation in CAM modalities, study designs, populations, intervention characteristics, and sleep outcome measures, a scoping review was considered the most appropriate approach to characterize the existing evidence and identify knowledge gaps, rather than to estimate pooled intervention effects. Accordingly, this review aimed to examine CAM interventions for sleep outcomes among perimenopausal women published between 2014 and 2024 and to highlight key patterns and gaps in the literature.

## Review

Methods

Study Design

This scoping review was conducted in accordance with the Preferred Reporting Items for Systematic Reviews and Meta-Analyses Extension for Scoping Reviews (PRISMA-ScR) [28]. A systematic search was undertaken to identify studies examining CAM interventions for sleep disturbances among healthy perimenopausal women. Searches combined MeSH terms and keywords, such as (perimenopause OR perimenopausal) AND (sleep OR insomnia) AND (complementary therapies OR acupuncture OR yoga). The full search strategies are provided in the Appendices.

The review was restricted to full-text primary research studies published in peer-reviewed sources to ensure sufficient methodological and outcome information for data charting. Gray literature was therefore not systematically searched. Eligibility was otherwise unrestricted, apart from publication date (2014-2024), English language, and the requirement that studies report sleep-related outcomes. The review aimed to map and describe the existing evidence, with particular attention to intervention types, study characteristics, and reported sleep-related outcomes. As the objective of this review was to map the evidence rather than evaluate intervention effectiveness, a formal methodological quality appraisal of the included studies was not performed.

Eligibility Criteria

Studies were eligible if they enrolled healthy midlife perimenopausal women aged approximately 40-60 years, reflecting the typical age range for the perimenopausal transition. Studies involving participants diagnosed with chronic medical conditions (e.g., kidney disease, cardiovascular disease, diabetes, autoimmune disorders, or other major systemic illnesses) were excluded unless data for healthy participants were reported separately. Health status was determined according to study-specific or established clinical criteria. Eligible interventions included CAM approaches, such as mind-body practices, manual therapies, herbal remedies, and other non-pharmacologic strategies. Studies were required to report at least one sleep-related outcome (e.g., sleep quality, insomnia, or sleep duration) assessed using validated instruments or clearly defined measures.

Only primary research designs were included, such as randomized controlled trials (RCTs), quasi-experimental studies, observational studies, qualitative studies, and case series. Secondary literature (e.g., narrative reviews, systematic reviews, meta-analyses), study protocols, editorials, commentaries, and conference abstracts were excluded. Publications were limited to English-language studies published between 2014 and 2024. Studies were also excluded if they focused exclusively on premenopausal or postmenopausal populations, evaluated pharmacologic or hormone-based interventions, or did not report sleep-related outcomes. Given the scoping nature of the review and the substantial heterogeneity in study designs, CAM modalities, intervention characteristics, populations, and sleep outcome measures, no meta-analysis or statistical pooling was undertaken. Findings were synthesized descriptively to map the characteristics of the evidence and identify patterns in the reported sleep outcomes, rather than to estimate pooled intervention effects.

Information Sources and Search Strategy

A comprehensive literature search was conducted across five electronic databases: PubMed, CINAHL (via EBSCOhost), Scopus, the Cochrane Library, and Web of Science. Searches covered publications from January 2014 through June 2024 and were limited to English-language studies involving human participants. The search strategies combined controlled vocabulary (where applicable) and free-text keywords related to perimenopause, CAM interventions, and sleep outcomes, with database-specific adaptations.

Screening and Selection

**Figure 1:**
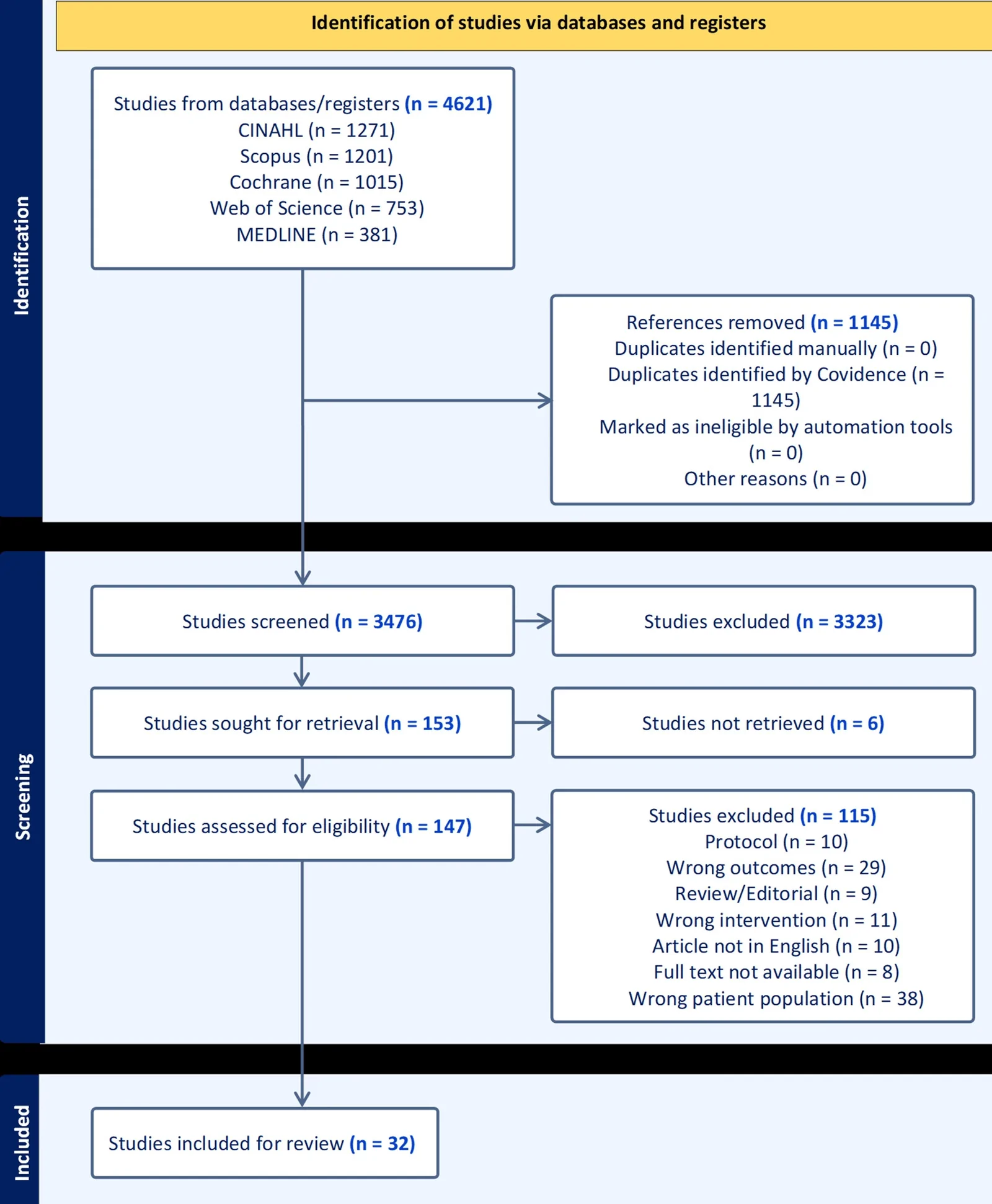
PRISMA diagram depicting article selection PRISMA: Preferred Reporting Items for Systematic Reviews and Meta-Analyses

All records retrieved from the database searches were imported into Covidence (Veritas Health Innovation, Melbourne, Australia), which was used for reference management, automatic de-duplication, title and abstract screening, and full-text review. Duplicate records were identified and removed automatically by the platform before screening. A team of four reviewers (ARB, KM, MMM, and AM) independently screened the records and extracted data. During the title and abstract screening phase, records were assessed for potential eligibility based on predefined criteria. Full-text screening was subsequently conducted by all four reviewers to identify the final set of eligible papers using the study’s inclusion and exclusion criteria. Discrepancies in eligibility assessment were resolved through discussion among all reviewers until consensus was achieved. The screening and study selection process was presented using the PRISMA 2020 flow diagram (Figure 1), adapted from Page et al. [29]. The diagram presents the number of records identified, screened, excluded, and included at each stage of the review. Extracted data were summarized in tables to facilitate a descriptive synthesis.

Data Charting, Synthesis and PRISMA-ScR Compliance

A standardized data extraction form was developed in accordance with PRISMA-ScR guidelines. For each included study, data were collected on publication characteristics, study design, sample demographics, country of origin, and CAM intervention type. Sleep-related outcome measures, including the Pittsburgh Sleep Quality Index (PSQI), Insomnia Severity Index (ISI), and other validated instruments, were recorded along with key findings. To facilitate descriptive synthesis, sleep outcomes were categorized as improved, unchanged, or worsened, based on statistical significance and direction of effect. This classification was used descriptively to map patterns across the literature and was not intended to represent a pooled estimate of intervention effectiveness.

Following completion of screening and consensus discussions, a total of 32 studies were included in the final synthesis. Data were synthesized descriptively to map the scope and nature of the evidence. Studies were grouped according to CAM intervention type, direction of sleep-related outcomes, and study design characteristics. Narrative summaries, summary tables, and descriptive figures were used to illustrate patterns and gaps within the existing literature. In accordance with best-practice standards, this review followed the PRISMA-ScR checklist to ensure transparency and completeness (Table 1).

**Table 1:** PRISMA-ScR checklist ^*^Where sources of evidence (see second footnote) are compiled from, such as bibliographic databases, social media platforms, and websites. ^†^A more inclusive/heterogeneous term used to account for the different types of evidence or data sources (e.g., quantitative and/or qualitative research, expert opinion, and policy documents) that may be eligible in a scoping review as opposed to only studies. This is not to be confused with information sources (see first footnote). ^‡^The frameworks by Arksey and O’Malley [6] and Levac et al. [7] and the JBI guidance [4,5] refer to the process of data extraction in a scoping review as data charting. ^§^The process of systematically examining research evidence to assess its validity, results, and relevance before using it to inform a decision. This term is used for items 12 and 19 instead of "risk of bias" (which is more applicable to systematic reviews of interventions) to include and acknowledge the various sources of evidence that may be used in a scoping review (e.g., quantitative and/or qualitative research, expert opinion, and policy documents) Adapted from: Tricco et al. [30] PRISMA-ScR: Preferred Reporting Items for Systematic Reviews and Meta-Analyses Extension for Scoping Reviews; JBI: Joanna Briggs Institute

Section	Item	PRISMA-ScR checklist item
Title		
Title	1	Identify the report as a scoping review
Abstract		
Structured summary	2	Provide a structured summary that includes (as applicable): background, objectives, eligibility criteria, sources of evidence, charting methods, results, and conclusions that relate to the review questions and objectives
Introduction		
Rationale	3	Describe the rationale for the review in the context of what is already known. Explain why the review questions/objectives lend themselves to a scoping review approach
Objectives	4	Provide an explicit statement of the questions and objectives being addressed with reference to their key elements (e.g., population or participants, concepts, and context) or other relevant key elements used to conceptualize the review questions and/or objectives
Methods		
Protocol and registration	5	Indicate whether a review protocol exists; state if and where it can be accessed (e.g., a Web address); and if available, provide registration information, including the registration number
Eligibility criteria	6	Specify characteristics of the sources of evidence used as eligibility criteria (e.g., years considered, language, and publication status) and provide a rationale
Information sources^*^	7	Describe all information sources in the search (e.g., databases with dates of coverage and contact with authors to identify additional sources), as well as the date the most recent search was executed
Search	8	Present the full electronic search strategy for at least 1 database, including any limits used, such that it could be repeated
Selection of sources of evidence^†^	9	State the process for selecting sources of evidence (i.e., screening and eligibility) included in the scoping review
Data charting process^‡^	10	Describe the methods of charting data from the included sources of evidence (e.g., calibrated forms or forms that have been tested by the team before their use, and whether data charting was done independently or in duplicate) and any processes for obtaining and confirming data from investigators
Data items	11	List and define all variables for which data were sought and any assumptions and simplifications made
Critical appraisal of individual sources of evidence^§^	12	If done, provide a rationale for conducting a critical appraisal of included sources of evidence; describe the methods used and how this information was used in any data synthesis (if appropriate)
Synthesis of results	13	Describe the methods of handling and summarizing the data that were charted
Results		
Selection of sources of evidence	14	Give numbers of sources of evidence screened, assessed for eligibility, and included in the review, with reasons for exclusions at each stage, ideally using a flow diagram
Characteristics of sources of evidence	15	For each source of evidence, present characteristics for which data were charted and provide the citations
Critical appraisal within sources of evidence	16	If done, present data on critical appraisal of included sources of evidence (see item 12)
Results of individual sources of evidence	17	For each included source of evidence, present the relevant data that were charted that relate to the review questions and objectives
Synthesis of results	18	Summarize and/or present the charting results as they relate to the review questions and objectives
Discussion		
Summary of evidence	19	Summarize the main results (including an overview of concepts, themes, and types of evidence available), link to the review questions and objectives, and consider the relevance to key groups
Limitations	20	Discuss the limitations of the scoping review process
Conclusions	21	Provide a general interpretation of the results with respect to the review questions and objectives, as well as potential implications and/or next steps
Funding		
Funding	22	Describe sources of funding for the included sources of evidence, as well as sources of funding for the scoping review. Describe the role of the funders of the scoping review

Results

The literature search across five databases identified 4,621 records, of which 32 studies met the inclusion criteria. The included studies were conducted primarily in China (n = 13), followed by the United States (n = 4), Canada (n = 4), India (n = 3), and Turkey (n = 2), while the remainder were conducted in other countries. Of the 32 included studies, 25 were RCTs. Whole medical systems (mainly TCM/acupuncture, n = 11) and biologically based practices (n = 11) were the most frequently represented CAM categories. Most studies (n = 26) reported improvements in sleep, most commonly assessed using the PSQI, although effect sizes and statistical significance varied widely.

**Figure 2:**
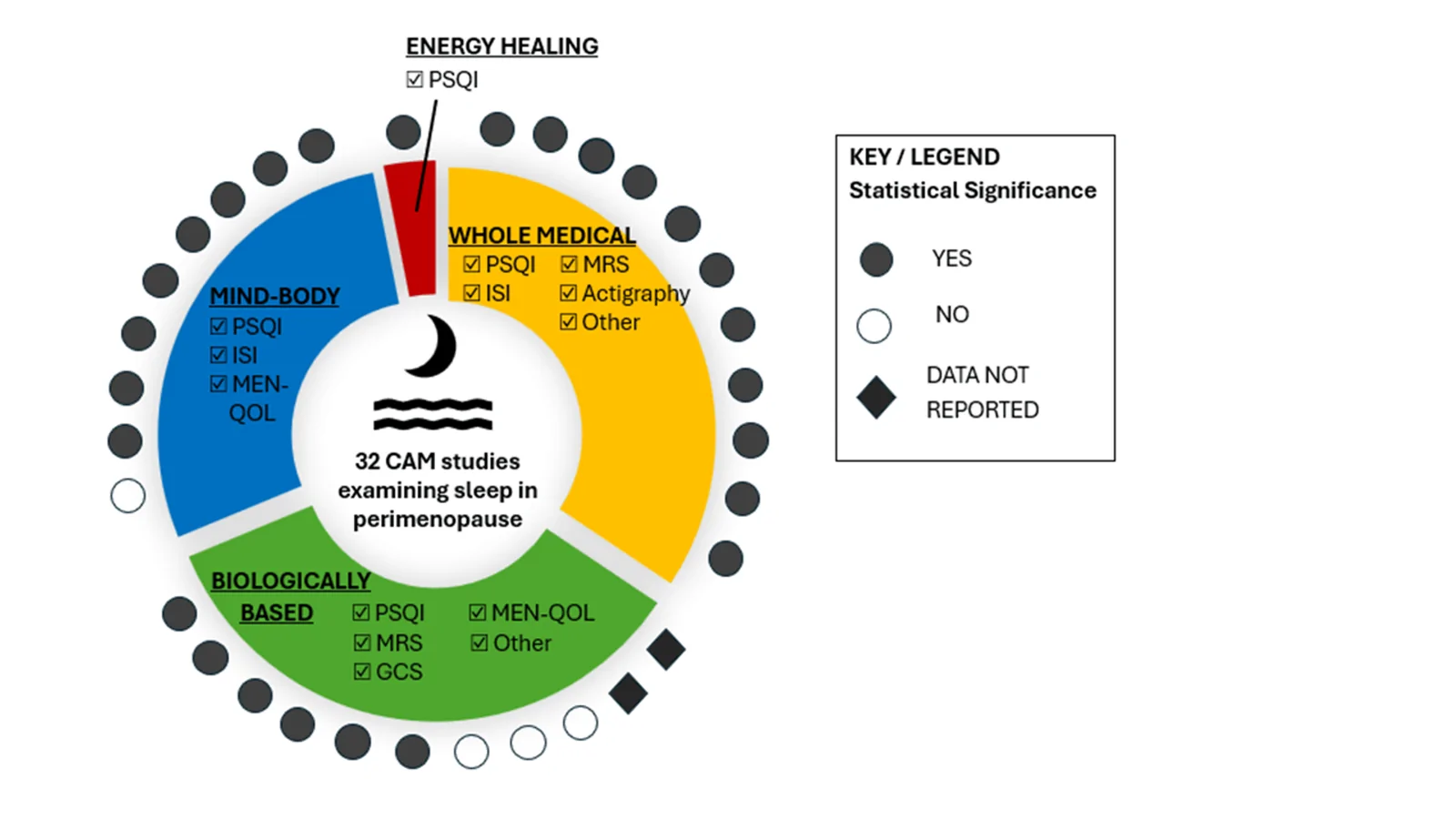
Distribution of CAM interventions by sleep outcomes in perimenopausal women CAM: complementary and alternative medicine; PSQI: Pittsburgh Sleep Quality Index; ISI: Insomnia Severity Index; MEN-QOL: Menopause-Related Quality of Life; MRS: Menopause Rating Scale; GCS: Greene Climacteric Scale

Across the domains of the National Cancer Institute’s classification of CAM, interventions were identified and evaluated for their effects on sleep among perimenopausal women (Figure 2). The inner segments represent the major CAM categories, while checkmarks within each segment denote the sleep-related outcome instruments used. Small peripheral markers arranged along the outer ring represent individual studies and indicate the statistical significance of sleep outcomes, as shown in the legend. Filled circles denote statistically significant improvement, open circles denote no statistically significant change, and diamonds indicate that data were not reported.

Whole Medical Systems/Traditional Chinese Medicine-Based Approaches

Eleven studies examined interventions classified as Whole Medical Systems, including wake and light therapy, moxibustion, and a range of acupuncture-based approaches. These approaches encompassed standard acupuncture, hand acupuncture, acupuncture combined with herbal decoctions, Yin-Yang-adjusted acupuncture, electroacupuncture (EA), and kidney-tonifying and mind-calming acupuncture. Across studies, acupuncture-based interventions, EA, and moxibustion were consistently associated with statistically significant improvements in sleep, as assessed using multiple validated sleep-related instruments. Four studies reported that acupuncture alone was associated with significant improvements in sleep outcomes. Li and Zhu [31] and Tang et al. [32] demonstrated improvements using the PSQI, while Fu et al. [33] reported significant improvements using both the PSQI and the ISI. Avis and colleagues [34] similarly observed significant improvements across multiple measures, including the PSQI, ISI, Women’s Health Questionnaire (WHQ), and PROMIS Sleep Disturbance scale.

Additional studies evaluated acupuncture in combination with other therapeutic components. Tan et al. in 2015 [35] reported significant improvements in sleep using the SPIEGEL scale following acupuncture combined with an herbal decoction. Yang et al. in 2024 [36] also demonstrated significant sleep improvements using the PSQI and ISI following kidney-tonifying and mind-calming acupuncture, while Bao et al. [37] reported improvements using the Menopause Rating Scale (MRS) following Yin-Yang-adjusted acupuncture. Three studies specifically evaluated EA, and all reported significant improvements in sleep as measured by the PSQI. The remaining study in this category, conducted by Feng et al. in 2022 [38], demonstrated that moxibustion was associated with improved sleep outcomes, also assessed using the PSQI.

Biologically Based Therapies

Among the eleven studies examining biologically based practices, eight reported improvements in sleep outcomes, with six demonstrating statistically significant improvements. Two studies evaluated soy-based interventions, with both soy germ extract and soy isoflavones associated with improvements in sleep; however, only soy germ extract demonstrated a statistically significant reduction in sleep difficulties [39,40]. Across this domain, a range of herbal and nutraceutical interventions were investigated, including Chinese herbal medicine (CHM) granules, fenugreek seed extract, Nu-femme, purified cytoplasm of pollen, Gengnianchun formula, and herbal formulations containing black currant extract. Statistically significant improvements in sleep were reported for CHM granules using the PSQI [41], fenugreek seed extract using the insomnia subscale of the MRS [42], purified cytoplasm of pollen using the Greene Climacteric Scale (GCS) [43], and Nu-femme using both the MRS and Menopause-Specific Quality of Life Questionnaire (MENQOL) [44].

In contrast, the herbal Gengnianchun formula granules [45] and black currant-based herbal formulations [46] did not demonstrate improvements in sleep outcomes, as assessed by the PSQI and the Oguri-Shirakawa-Azumi Sleep Inventory, respectively. Additional biologically based CAM interventions included Amberen, JuicePLUS+, and cannabis. While Amberen led to significant improvements in sleep symptoms on the GCS [47], JuicePLUS+ was associated with modest improvements in sleep problems on the MRS [48]. A cross-sectional study further reported fewer self-reported sleep difficulties among cannabis users than among non-users [49].

Mind-Body Practices

Mind-body practices were represented in nine studies and included yoga-based interventions, MBSR, progressive muscle relaxation, Benson’s relaxation therapy, and hypnosis. Three studies evaluated yoga-based interventions. Sijna and Shobhana [50] reported that a clinical yoga package significantly improved sleep as measured by the MENQOL. Similarly, Newton et al. [51] observed significant improvements in sleep using both the ISI and the PSQI, while Guthrie et al. [52] reported significant improvements using the PSQI alone. Progressive muscle relaxation was examined in two studies, both of which reported improvements in sleep outcomes. Augoulea et al. [53] reported improvements using the PSQI, while Aksu and Erenel [54] observed significant improvements using the Insomnia Rating Scale (IRS).

Ismail and colleagues demonstrated that Benson’s relaxation therapy combined with physical activity [55] was associated with improvements in sleep, as assessed by the PSQI. Although physical activity alone was not classified as CAM for this review, this study was included because the primary intervention incorporated a recognized mind-body modality. Two studies examined MBSR using the PSQI, with both reporting improvements in sleep; however, statistical significance was observed in only one study [56], while the other reported non-significant improvements [57]. Finally, Elkins et al. demonstrated that hypnosis was associated with improved sleep outcomes, as measured by the PSQI [58].

Energy Therapies and Manipulative and Body-Based Practices

The energy therapies category was represented by a single study, in which therapeutic touch was associated with a statistically significant improvement in sleep outcomes, as assessed using the PSQI [59]. No studies were identified within the manipulative and body-based practices category.

Discussion

Overview of the Evidence 

This scoping review aimed to describe and characterize the existing literature on CAM interventions for sleep disturbances during perimenopause, with particular attention to sleep assessment measures. Although interest in this area has grown in recent years, the overall body of evidence remains limited. The available literature demonstrates considerable heterogeneity and uneven representation across intervention modalities, study populations, and outcome measures. Notably, relatively few studies have prioritized sleep as a primary endpoint or focused specifically on women in the perimenopausal stage. The sections that follow synthesize how sleep outcomes, intervention characteristics, and perimenopausal symptoms were characterized across 32 studies and highlight methodological and conceptual considerations relevant to future integrative medicine research. The absence of a formal quality appraisal limits our ability to assess the strength or certainty of the evidence.

Across the included studies, sleep was evaluated alongside a broader constellation of perimenopausal symptoms, including vasomotor symptoms, psychological distress, including anxiety and depression, fatigue, and quality-of-life impairment. Studies conceptualized sleep as a biopsychosocial phenomenon shaped by several factors, including neuroendocrine, behavioral, and emotional influences. This is consistent with evidence that sleep disturbances during the perimenopausal phase are influenced by interacting biological, psychological, and social predictors, including hormonal changes, vasomotor symptoms, mood disturbance, and life stressors [60-62].

Patterns Across CAM Intervention Domains

Mind-body therapies represented a substantial portion of the mapped evidence, reflecting sustained research interest in interventions targeting stress regulation and psychophysiological processes relevant to sleep. These approaches included MBSR, structured stress-management programs, yoga-based interventions, relaxation therapies, such as progressive muscle relaxation and Benson’s relaxation therapy, and hypnosis [50-58]. Interventions were typically delivered in structured group-based or individual formats, with session frequency ranging from weekly to multiple sessions per week, often incorporating guided home practice using written materials, audio recordings, or instructor-led exercises to support skill acquisition and adherence outside the clinical setting. A recent meta-analysis reported findings consistent with the present review, demonstrating that non-pharmacological interventions were associated with improved sleep quality and reduced insomnia severity among perimenopausal women with sleep disturbances [63-66].

Across these mind-body studies, sleep outcomes were commonly assessed alongside measures of perceived stress, anxiety, depression, functional dyspepsia, and menopause-related quality of life, underscoring an integrative conceptualization of sleep disturbance within a broader emotional and psychosocial framework. Studies differed significantly in intervention duration and intensity, instructor training, and delivery modality (in-person, group-based, or hybrid formats). This variability not only reflects the diversity of mind-body intervention models, but also limits direct comparison across studies and highlights the challenges in synthesizing findings across various integrative approaches.

Interventions based on TCM constituted another major domain, with acupuncture being the most frequently examined practice. Acupuncture-based interventions included both traditional and contemporary TCM techniques. Older modalities, such as fire dragon pot moxibustion and auricular (ear) acupuncture, are rooted in long-standing Chinese medical theory and emphasize warming, tonification, and regulation of qi/energy through thermal stimulation and the use of small, mapped body regions, such as the ear, believed to correspond to whole-body functions. In contrast, more contemporary approaches, such as hand acupuncture and EA, represent modern adaptations of traditional principles designed to enhance treatment precision, standardization, and reproducibility. Consistent with the patterns identified in the present scoping review, a recent meta-analysis of randomized controlled trials reported that acupuncture significantly improved sleep quality and reduced insomnia severity among perimenopausal women, although variability across intervention protocols and outcome measures was noted [64].

These TCM studies were predominantly conducted in China and were commonly grounded in traditional diagnostic frameworks, including kidney deficiency, liver-kidney imbalance, or yin-yang disharmony, which informed the selection of acupuncture points, herbal composition, and treatment sequencing and duration. In several trials, interventions were semi-standardized, combining fixed treatment components with individualized adjustments based on symptom presentation or TCM pattern differentiation. While all modalities are grounded in TCM concepts, they differ in their methods of stimulation, degree of practitioner involvement, and suitability for controlled clinical research settings, which may contribute to variability in reported outcomes.

Several studies also employed multimodal CAM approaches, most commonly within TCM-based interventions, combining acupuncture with auricular therapy [67], moxibustion [38], herbal formulations [35,41,45], or psychotherapy [39]. Some trials incorporated behavioral or movement-based components [55], while others provided background herbal treatments to all study arms [68], reflecting a more pragmatic integrative practice. These combined therapeutic options attempted to address the interconnection between physical and emotional symptoms rather than focusing on sleep disturbances in isolation. Treatment protocols also varied widely in session frequency, duration, needle retention time, stimulation techniques, and total intervention length, contributing to substantial methodological variation. Outcome assessment varied across studies, and sleep outcomes were often measured alongside menopausal symptoms, psychological distress, and quality-of-life indicators.

The concentration of TCM-based trials within hospital settings in China raises important considerations regarding external validity, generalizability, and accessibility in other healthcare systems. Differences in cultural familiarity, reimbursement policies, and workforce availability may influence feasibility and sustainability in non-TCM-dominant contexts. Therefore, evaluating cross-cultural adaptation strategies and community-based delivery pathways will be essential for determining broader applicability and acceptance.

An expanding body of literature also examined dietary supplements, botanical extracts, and nutraceutical products as nonhormonal CAM options for perimenopausal symptom management. Interventions included soy isoflavones, soy germ extract, fenugreek seed extract, Labisia pumila and Eurycoma longifolia formulations, polyphenol-rich preparations, purified cytoplasm of pollen, Chinese herbal granules, multicomponent nutraceutical blends, and succinate-based supplements such as Amberen® [39-42,44,46-49]. Sleep outcomes were typically secondary endpoints assessed alongside vasomotor symptoms, mood, and overall menopausal symptom burden. Treatment components, dosing, and duration varied widely, highlighting both the diversity of available products and the lack of standardization within this research domain.

One observational study examined self-reported cannabis use among midlife women for menopause-related symptoms, including sleep disturbance [49]. This study highlights self-directed CAM use outside formal research settings and reveals a gap between real-world holistic practices and interventions commonly evaluated in controlled trials. As highlighted by Dahia et al., growing clinical and consumer interest in natural health products (NHPs) reflects their perceived potential to support sleep, mood, and stress regulation during midlife, despite variable levels of empirical support. While NHPs are increasingly considered relevant by midlife women, many NHPs lack rigorous scientific evidence supporting their efficacy and safety [69].

The widespread availability and consumer-driven use of supplements and NHPs introduce considerations related to product standardization, regulatory oversight, and informed decision-making. Inconsistent labeling, variable bioactive concentrations, and limited post-market evaluation may contribute to heterogeneous outcomes and potential safety risks, particularly among women using concurrent prescription therapies. Strengthening transparency in reporting, improving pharmacovigilance mechanisms, and supporting clinician-patient dialogue are critical to ensuring safe, informed, and evidence-based use across diverse populations.

The evidence base for energy therapies and manipulative and body-based practices was sparse, with only one study demonstrating improved sleep outcomes following therapeutic touch. This highlights a significant research gap, particularly given that some of these approaches are accessible in community and integrative care settings. The absence of rigorous evaluation restricts the assessment of effectiveness, safety, and equity of access and limits the ability to inform clinical recommendations or broader midlife health strategies. Targeted research is therefore needed to clarify whether these modalities warrant consideration within comprehensive, population-level approaches to sleep health during perimenopause.

Outcome Measurement Patterns

Across CAM modalities, sleep outcomes were predominantly assessed using self-reported instruments, most commonly the PSQI. Other measures included the ISI, MRS, GCS, Kupperman Index, SPIEGEL scale, PROMIS Sleep Disturbance scores, and the middle-aged and elderly versions of the Oguri-Shirakawa-Azumi sleep inventory. Sleep disorders and insomnia largely remain clinical diagnoses based on patients’ subjective complaints [9], which may partly explain the widespread reliance on self-reported instruments. Psychological and quality-of-life measures were also frequently included, reinforcing the multidimensional framing of sleep disturbance during perimenopause. Only a limited number of studies incorporated objective sleep assessments, such as actigraphy or polysomnography [33,68]. Although self-reported measures are low-burden, feasible for use in all settings, and capture lived experiences and changes that matter to patients, the infrequent use of objective measures limits our understanding of the underlying mechanisms governing physiological sleep processes during the midlife transition.

Greater integration of objective assessments may therefore strengthen measurement precision and improve comparability across studies and settings. From a population health perspective, consistent and validated outcome measurement is essential for monitoring trends, evaluating intervention effectiveness, and informing midlife health initiatives.

Population Characteristics and Contextual Gaps

Across the 32 included studies, participant samples primarily consisted of midlife women aged approximately 40-60 years, reflecting the typical age range of the perimenopausal transition. However, most studies were conducted in relatively homogeneous populations, with limited reporting of racial, ethnic, or socioeconomic diversity. A substantial proportion of the evidence, particularly studies examining TCM-based interventions such as acupuncture and moxibustion, originated from East Asia, whereas studies conducted in Europe, India, and North America more frequently focused on mind-body therapies, dietary supplements, and self-directed CAM use. This geographic distribution may reflect regional differences, cultural familiarity, and patient acceptance of various CAM modalities, as well as the degree of CAM integration into existing healthcare infrastructures [14,70-72]. The concentration of TCM-based trials in China raises important considerations regarding the external validity and generalizability of findings to other healthcare systems.

In addition, few studies explicitly examined how cultural context, health beliefs, access to care, or healthcare system differences may shape the acceptance and effectiveness of CAM interventions during perimenopause. Socioeconomic factors, including education, income, and insurance status, were inconsistently reported, and social determinants of health were rarely integrated into study design or analysis. This represents an important contextual gap, given that CAM use patterns, preferences, and barriers to use vary substantially across racial, ethnic, and socioeconomic groups [73].

Temporal Scope and Follow-Up

Most interventions were evaluated over short to medium durations, typically ranging from four to twelve weeks, and although some studies included follow-up assessments, long-term evaluation beyond six months was uncommon. As a result, relatively little is known about the long-term impact of observed changes in sleep and related symptoms throughout the broader perimenopausal transition. Furthermore, the absence of extended follow-up limits assessment of delayed effects, adherence patterns, and potential cumulative benefits or challenges associated with sustained CAM use. Insufficient longitudinal data also limit our understanding of how midlife sleep disturbances and their management may influence longer-term mental health, cardiometabolic risk, and healthcare utilization. Incorporating longer-term follow-up periods in future studies may better capture the temporal dynamics of sleep-related changes and integrative intervention use during midlife.

Reporting of safety and adverse events also varied substantially across CAM modalities and study designs. Among acupuncture and EA trials, several studies explicitly monitored and reported adverse events, which were generally described as mild and transient, including local pain, bruising, minor bleeding, or temporary discomfort at needle sites [33,34,36,68,74,75]. EA studies similarly reported short-term local or sensory effects related to stimulation, with no serious adverse events documented within the intervention periods [68,75]. In one study that combined EA with Luohua Anshen oral liquid, one patient experienced slight dizziness during the first acupuncture treatment [76]. However, the scope and detail of adverse event reporting were inconsistent, and safety outcomes were rarely defined as primary outcomes.

For dietary supplements, botanicals, and nutraceuticals, safety reporting was also highly variable. Some trials noted monitoring for adverse events and described mild gastrointestinal symptoms, headache, or transient discomfort associated with supplement use [39,40,42,44,47], whereas others provided limited to no explicit adverse event data despite relatively longer intervention durations [45,46,48]. Few studies addressed potential interactions with concurrent medications or hormonal therapies. From a public health perspective, inadequate monitoring of potential interactions has implications for medication safety, polypharmacy risk, and the prevention of population-level harm.

In contrast, mind-body practices, including MBSR and other stress-management programs, relaxation therapies, and hypnosis, rarely reported adverse events in detail [50-58]. When safety was addressed, these approaches were generally described as well tolerated; however, systematic monitoring and reporting of adverse events were uncommon. Limited safety reporting in this domain may reflect assumptions of low risk, yet the absence of structured surveillance constrains the ability to assess tolerability across diverse populations, delivery formats, and implementation contexts. Details of all included studies are presented in Tables 2-3.

**Table 2:** Description of CAM by study design BRT: Benson’s relaxation therapy; CAM: complementary and alternative medicine; CHM: Chinese herbal medicine; EA: Electroacupuncture; MBSR: mindfulness-based stress reduction; PCP: purified cytoplasm of pollen; PMR: progressive muscle relaxation; RCT: randomized controlled trial

Study	Country	Study design	Sample size	CAM classification and name	CAM Description
				Whole medical systems	CAM description
Tang et al. (2023) [32]	China	Case series	42	Acupuncture	Acupuncture applied at GV20, EX-HN1, GV16, EX-HN18, GV11, BL18, BL20, BL23, HT7, and SP6; de qi sensation obtained; needles retained for 30 minutes; electric stimulation applied between paired points (BL18–BL20, GV20–GV11). Conducted three times per week for 4 weeks
Fu et al. (2017) [33]	China	RCT	76	Acupuncture	Acupuncture performed by a licensed TCM practitioner using 0.25×40 mm needles inserted 10-30 mm at BL23, BL18, LR14, and GB25. Manual stimulation to elicit de qi sensation; needles retained for 20 min; 10 sessions over 3 weeks. The Sham group received Streitberger placebo needles at the same sites
Avis et al. (2016) [34]	USA	RCT	209	Acupuncture	Participants received up to 20 individualized acupuncture sessions over 6 months by licensed acupuncturists (8–33 years' experience). Points selected based on TCM diagnosis; de Qi sensation elicited when possible; optional electrical or manual stimulation allowed
Tan et al. (2015) [35]	China	RCT	126	Acupuncture + herbal decoction	4 weeks of daily acupuncture, which comprised moderate manipulation; retained for 30 minutes; manipulated every 10 minutes, given at various points over the body. “Acupoints: Taixi (Kidney 3), Sanyinjiao (Spleen 6), Baihui (Governor Vessel 20), Sishencong (Extra Head and Neck 1), Shenmen (Heart 7), Shenmai (Bladder 62), and Zhaohai (Kidney 6)
Yang et al. (2024) [36]	China	RCT	90	Kidney-tonifying and Mind-calming acupuncture	Acupuncture administered at Baihui (GV20), Shenshu (BL23), Taixi (KI3), and Anmian (Extra) three times weekly for four weeks. Balanced reinforcing and reducing technique used after de qi sensation; needles were retained for 30 minutes per session. Sham acupuncture performed with superficial needling (1–2 mm)
Bao et al. (2015) [37]	China	RCT	60	Yin–Yang-adjusted acupuncture	Acupuncture based on Yin-Yang balance principles administered over 8 weeks; compared with placebo acupuncture
Feng et al. (2022) [38]	China	RCT	70	Moxibustion	The intervention group additionally received fire dragon pot moxibustion for 10 weeks to enhance sleep and mood. The control group received auricular point seed burying
Li and Zhu (2014) [67]	China	RCT	66	Acupuncture	Acupuncture - 4 weeks - once every other day, three days a week. The needles at Baihui (Governor Vessel 20) and Shenting (Governor Vessel 24) were connected to an electroacupuncture instrument with a sparse-dense wave
Zhao et al. (2019) [68]	China	RCT	48	Hand EA	Hand acupuncture therapy applied every other day, 3× per week for 8 weeks; sham group received placebo stimulation
Li et al. (2020) [75]	China	RCT	84	EA	Electroacupuncture at 10 acupoints (GV20, GV24, GV29, CV6, CV4, EX-HN22, SP6, HT7 + syndrome-specific points GV4, BL23, KI3, KI7) for 30 min sessions, 18 sessions, over 8 weeks; continuous electrical stimulation (2.5 Hz, 4–5 mA). Sham used Streitberger non-invasive needles without current. PSQI at the end of 8 weeks was the primary outcome
Zhao et al. (2019) [76]	China	RCT	66	EA	Sterilized disposable needles (0.25 mm in diameter and 25 mm in length) were inserted subcutaneously into Sishencong (EX-HN 1), Baihui (GV 20), Shenting (GV 24), and Benshen (GB 13), while Shenmai (BL 62) and Zhaohai (KI 6) were inserted perpendicularly.
					All of the needles were retained for 30 min. The EA treatment was performed once every other day, three days a week. A total of a 4-week treatment was given
					During the period - in both the EA and control groups, patients were given one dose of Luohua Anshen oral liquid 30 min after breakfast and dinner, respectively, and two doses of it 30 min before sleep per night, for 4 weeks in total
				Biologically based practices	
Imhof et al. (2018) [39]	Austria, Romania, Germany	RCT	192	Soy germ extract	Soy germ extract (100 mg isoflavone glycosides) daily or with placebo for 12 weeks, followed by 12 weeks of open treatment with soy
Ahsan and Mallick (2017) [40]	India	Non-RCT	50	Soy isoflavone	Patients were prescribed 100 mg soy isoflavone supplements per day for 12 weeks by the treating gynecologist for menopausal symptoms
					To ensure that the diet did not contain any other soy-based foods, they were given a list of foods to avoid during the three months of therapy
					Patients who had previous treatment with hormonal
					treatment or soybean-derived products in the previous 12 months were excluded from the study
					Menopausal Rating Scale questionnaire was used before starting therapy and after 12 weeks of therapy with 100 mg soy isoflavones
Cao et al. (2019) [41]	China	RCT	122	CHM granules	Modified “Erxian Decoction” (Tonifying Kidney–Soothing Liver formula); composed of traditional herbs including Epimedium brevicornum, Curculigo orchioides, Morinda officinalis, Anemarrhena asphodeloides, Phellodendron amurense, Angelica sinensis, Ligusticum chuanxiong, Paeonia lactiflora, etc.; taken orally as granules twice daily for 12 weeks alongside conventional hormone therapy
Khanna et al, (2020) [42]	India	RCT	48	Fenugreek seed extract (FHE; FenuSMART®)	Standardized hydro-ethanolic extract of fenugreek seeds containing 10.3% protodioscin, 3.1% trigonelline, and 42.6% total saponins in a 3:1 ratio of protodioscin to trigonelline. Participants received 250 mg of capsules twice daily for 42 days. The extract exhibits estrogenic and serotonergic activity
Lello et al. (2022) [43]	Italy	Other quantitative	108	PCP	PCP is a nonhormonal herbal remedy administered to manage vasomotor, mood, and sleep disturbances during menopause without hormone therapy. Participants were evaluated at baseline and after 3 months of treatment
Chinnappan et al. (2020) [44]	Canada	RCT	119	Nu-femme™ (combination of Labisia pumila (SLP+®) and Eurycoma longifolia (Physta®))	Two capsules daily after breakfast for 24 weeks (total = 400 mg L. pumila + 100 mg E. longifolia). Standardized water extracts prepared under GMP. Assessed hot flushes, MRS, MENQOL, hormones, and lipid profiles
Zhang et al. (2020) [45]	China	RCT	98	Gengnianchun - granulated	Each dose of I-GNC formula contained 97 g of crude herbal materials extracted into 11.6 g of granules and packaged in an aluminum foil sachet with a medicinal composite membrane inside. The placebo contained 10% I-GNC granules and other inactive ingredients. Participants were instructed to drink one sachet of granules dissolved in 100 mL of warm water once daily. A 4-week supply was dispensed on each treatment visit
Ohtsuka et al. (2024) [46]	Japan	RCT	59	Black currant extract	Participants ingested four tablets per day (400 mg total Cassis polyphenol, containing anthocyanins from blackcurrant extract AC10). Each dose was taken after breakfast and before bedtime for 4 weeks. Anthocyanins such as delphinidin-3-rutinoside and cyanidin-3-rutinoside were the primary active components
Kachko et al. (2024) [47]	Russia	RCT	105	Amberen	Daily Amberen® (two capsules 200 mg, succinate base + vitamin E) + Smart B® (one capsule 166 mg; vitamins B1, B2, B6, B9, B12), taken with breakfast for 90 days. Targets energy metabolism and stress resilience via succinate-mediated mitochondrial regulation and vitamin B support
Siebler et al.(2016) [48]	Germany	Other qualitative	28	JuicePLUS+	Participants took two daily capsules of JuicePLUS+, an encapsulated powder concentrate containing extracts from 30 fruits, vegetables, and berries enriched with natural vitamins and antioxidants. Each capsule included a standardized mixture of fruit, vegetables, and berry blends aimed at supporting metabolism and reducing oxidative stress. With a baseline, 8 weeks, and 16 weeks
Babyn et al.(2023) [49]	Canada	Cross- sectional	1485	Cannabis	The term ‘cannabis’ was used as an all-encompassing term including any form of cannabis or marijuana and any components (including THC or CBD) derived from the cannabis plant. Other forms of CAM reported: exercise and yoga, mindfulness and meditation, natural health products (NHPs)
				Mind-body practices	
Sijna and Shobhana (2021) [50]	India	RCT	40	Yoga	Yoga sessions conducted daily for 60 days, including postures, breathing exercises, and relaxation components designed to improve menopausal symptoms
Newton et al. (2014) [51]	USA	RCT	249	Yoga	Twelve 90-min weekly classes with “cooling” breathing (pranayama), 11–13 restorative poses (forward bends, inversions, twists, relaxation), and Yoga Nidra meditation; daily 20-min home practice using DVD/CD guidance
Guthrie et al. (2018) [52]	USA and Canada	RCT	546	Yoga	Yoga: a structured 12-week yoga program with breathing, stretching, and relaxation aimed at improving sleep and reducing vasomotor symptoms.
Augoulea et al. (2021) [53]	Greece	Pilot RCT	61	PMR	The intervention group received 8 weeks of training on the techniques of diaphragmatic breathing and progressive muscle relaxation, provided via an audio compact disc (total duration of 25 minutes: 10 minutes diaphragmatic breathing, and 15 minutes progressive muscle relaxation)
Aksu et al. (2022) [54]	Turkey	RCT	90	PMR	Audio Records of Relaxation Techniques were converted to digital audio and delivered to the participants. Each muscle group is tensed while taking a deep breath, and then the muscle groups are relaxed while gradually releasing the breath. The relaxation period lasts for 10–20 seconds before the next muscle group is tensed. Participants were asked to practice progressive muscle relaxation every day of the week or at least 4 days a week. The Women’s Health Initiative Insomnia Rating Scale was used, and a total score of 10 or above indicates that the individual suffers from insomnia. This study calculated the scores during the 7-day baseline period as well as at the end of the 4th and 8th week of intervention
Ismail et al. (2022) [55]	Egypt	RCT	60	BRT + physical activity	40-minute Benson’s relaxation therapy (BRT) (diaphragmatic breathing and progressive muscle relaxation applied for 20 minutes in the morning and evening)
Xiao et al. (2020) [56]	China	Other qualitative	121	MBSR	Eight-week structured MBSR program (twice weekly, 1 h sessions) led by certified trainers. Modules: body relaxation, body scan, breathing meditation, visualization, tension release, and compassion-based mindfulness. Participants were encouraged to self-practice 40 min/day with mobile app reminders
Gordon et al. (2020) [57]	Canada	RCT	104	MBSR	The 8-week MBSR program included group meditation, mindful yoga, relaxation, and home-based practice (45 min/day). Delivered by certified MBSR instructors with high fidelity
Elkins et al. (2021) [58]	USA	RCT	90	Hypnosis	Two different “doses” (three vs. five) of contact with research therapists and two delivery methods
					(in-person hypnosis sessions delivered by a therapist or contact via telephone only), with at-home practice with audio recordings for all groups
				Energy therapies	
Yalcinkaya and Gozuyesil (2023) [59]	Turkey	RCT	48	Therapeutic touch	The therapeutic touch practice was between 18:00 and 20:00; the intervention group administered therapeutic touch for 10 minutes for 5 successive days
					The procedures implemented in line with the Therapeutic Touch Practice Procedure included the following steps: centering, assessing, rebalancing, reassessing, reexamining, grounding, and closure

**Table 3:** CAM by sleep index and outcome BRT: Benson’s relaxation therapy; CAM: complementary and alternative medicine; FHE: standardized fenugreek extract; GCS: Greene Climacteric Scale; ISI: Insomnia Severity Index; MBSR: mindfulness-based stress reduction; MENQOL: Menopause-Specific Quality of Life; MRS: Menopause Rating Scale; OSA-MA: Oguri-Shirakawa-Azumi Sleep Inventory; PMR: progressive muscle relaxation; PROMIS-SD: Patient-Reported Outcomes Measurement Information System-Sleep Disturbance; PSQI, Pittsburgh Sleep Quality Index; TCMSSS: Traditional Chinese Medicine Sleep Syndrome Scale; WHQ: Women’s Health Questionnaire

Study	CAM Name	Outcome Index	Improved Sleep	Statistically Significant
	Whole medical systems			
Tang et al. (2023) [32]	Acupuncture	PSQI	Yes	Yes
Fu et al. (2017) [33]	Acupuncture	PSQI	Yes	Yes
Avis et al. (2016) [34]	Acupuncture	PSQI	Yes	Yes
Tan et al. (2015) [35]	Acupuncture + herbal decoction	SPIEGEL scale	Yes	Yes
Yang et al. (2024) [36]	Kidney-tonifying and mind-calming acupuncture	PSQI	Yes	Yes
		ISI		
Bao et al. (2015) [37]	Yin–Yang-adjusted acupuncture	MRS	Yes	Yes
Feng et al. (2022) [38]	Moxibustion	PSQI	Yes	Yes
		Total sleep time		
		Sleep latency		
Zhao et al. (2019) [68]	Hand electroacupuncture	PSQI	Yes	Yes
Li et al. (2020) [75]	Electroacupuncture	PSQI	Yes	Yes
Zhao et al. (2019) [76]	Electroacupuncture	PSQI	Yes	Yes
		TCMSS		
	Biologically based practices			
Imhof et al. (2018) [39]	Soy germ extract	GCS	Yes	Yes
Ahsan and Mallick (2017) [40]	Soy isoflavone	MRS	Yes	No
Cao et al. (2019) [41]	Chinese herbal medicine (CHM) granules	PSQI	Yes	Yes
Khanna et al. (2020) [42]	Fenugreek seed extract (FHE; FenuSMART®)	MRS	Yes	Yes
Lello et al. (2022) [43]	Purified cytoplasm of ollen (PCP)	GCS	Yes	Yes
Chinnappan et al. (2020) [44]	Nu-femme™ (Labisia pumila (SLP+®) and Eurycoma longifolia (Physta®)	MRS	Yes	Yes
Zhang et al. (2020) [45]	Gengnianchun formula granules	PSQI	No	No
Ohtsuka et al. (2024) [46]	Black currant extract	OSA-MA	No	No
Kachko et al. (2024) [47]	Amberen	GCS	Yes	Yes
Siebler et al. (2016) [48]	JuicePLUS+	MRS	Yes	Data not reported
Babyn et al. (2023) [49]	Cannabis	Difficulty sleeping	Data not reported	Data not reported
	Mind-body practices			
Sijna and Shobhana (2021) [50]	Yoga	MENQOL	Yes	Yes
Newton et al. (2014) [51]	Yoga	PSQI	Yes	Yes
		ISI		
Guthrie et al. (2018) [52]	Yoga	PSQI	Yes	Yes
Augoulea et al. (2021) [53]	PMR	PSQI	Yes	Yes
Aksu et al. (2022) [54]	PMR	ISI	Yes	Yes
Ismail et al. (2022) [55]	BRT + physical activity	PSQI	Yes	Yes
Xiao et al. (2020) [56]	MBSR	PSQI	Yes	Yes
Gordon et al. (2020) [57]	MBSR	PSQI	Yes	No
Elkins et al. (2021) [58]	Hypnosis	PSQI	Yes	Yes
	Energy therapies			
Yalcinkaya and Gozuyesil (2023) [59]	Therapeutic touch	PSQI	Yes	Yes

Reporting of Safety and Adverse Events across CAM Modalities

Reporting of safety and adverse events varied substantially across CAM modalities and study designs. Among acupuncture and EA trials, several studies explicitly monitored and reported adverse events, which were generally described as mild and transient, including local pain, bruising, minor bleeding, or temporary discomfort at needle sites [33,34,36,68,75]. EA studies similarly reported short-term local or sensory effects related to stimulation, with no serious adverse events documented within the intervention periods [68,75]. However, the scope and detail of adverse event reporting were inconsistent, and safety outcomes were rarely prespecified as primary outcomes.

For dietary supplements, botanicals, and nutraceuticals, safety reporting was more heterogeneous. Some trials noted monitoring for adverse events and described mild gastrointestinal symptoms, headache, or transient discomfort associated with supplement use [39,40,42,44,47], whereas others provided limited or no explicit adverse event data despite relatively longer intervention durations [45,46,48]. Few studies addressed potential interactions with concurrent medications or hormonal therapies. From a public health perspective, inadequate monitoring of potential interactions has implications for medication safety, polypharmacy risk, and the prevention of population-level harm.

In contrast, mind-body practices, including MBSR, yoga, relaxation therapies, stress-management programs, and hypnosis, rarely reported adverse events in detail [50-58]. When safety was addressed, these approaches were generally described as well tolerated; however, systematic monitoring and reporting of adverse events were uncommon. Limited safety reporting in this domain may reflect assumptions of low risk, yet the absence of structured surveillance constrains the ability to assess tolerability across diverse populations, delivery formats, and implementation contexts.

Overall, heterogeneous and incomplete safety reporting represents a notable evidence gap. Greater standardization and transparency in safety reporting, including clear definitions, routine monitoring protocols, and documentation of medication interactions, are essential. Strengthening safety surveillance will also help support risk-benefit assessment, enhance external validity, and ensure that recommendations for perimenopausal populations are both effective and safe at the population level.

## Conclusions

This review highlights an expanding body of research examining CAM therapies for perimenopausal sleep disturbances. Although improvements in sleep outcomes were reported across several CAM interventions, findings should be interpreted cautiously given substantial heterogeneity in study designs, intervention types, sample characteristics, and sleep outcome measures. Consequently, the available evidence does not support firm conclusions about the effectiveness of CAM interventions for improving sleep during the perimenopausal transition. The geographic concentration of the evidence, particularly the predominance of studies conducted in China, further limits its generalizability and cultural applicability across populations with differing healthcare systems, CAM traditions, cultural beliefs, and access to care. Sleep during the perimenopausal transition reflects a complex interplay of physiological, psychosocial, and pre-existing health factors, underscoring the need for individualized approaches to care.

From a clinical and population health perspective, routine sleep screening and early supportive interventions in primary and community care settings may help address the broader mental and cardiometabolic health consequences of poor sleep. However, translating CAM evidence into practice requires a greater understanding of its applicability across diverse populations and healthcare contexts. Future research should prioritize methodologically rigorous studies with greater geographic and population diversity, including women experiencing structural barriers to care, chronic stress, adverse life experiences, and cumulative health disadvantage. Such research is needed to clarify which CAM approaches, if any, are effective, for whom, and under what circumstances, while strengthening the equity, cultural relevance, and global applicability of the evidence for sleep health during the menopausal transition.

## Appendices

Appendix A - supplementary file 1 - search strings

PUBMED

(("Perimenopause"[MeSH] OR perimenopausal OR "menopausal transition" OR menopause OR "menopausal women" OR "midlife women") AND ("Complementary Therapies"[MeSH] OR "Alternative Medicine"[MeSH] OR CAM OR yoga OR acupuncture OR mindfulness OR meditation OR aromatherapy OR "herbal medicine" OR supplements OR exercise OR "behavioral therapy") AND ("Sleep"[MeSH] OR sleep OR insomnia OR "vasomotor symptoms" OR "hot flashes" OR mood OR depression OR anxiety OR "quality of life" OR fatigue)) 

*CINAHL* 

(("Perimenopause" OR perimenopausal OR "menopausal transition" OR menopause OR "menopausal women" OR "midlife women") AND ("Complementary Therapies" OR "Alternative Medicine" OR CAM OR yoga OR acupuncture OR mindfulness OR meditation OR aromatherapy OR "herbal medicine" OR supplements OR exercise OR "behavioral therapy") AND ("Sleep" OR sleep OR insomnia OR "vasomotor symptoms" OR "hot flashes" OR mood OR depression OR anxiety OR "quality of life" OR fatigue)) 

SCOPUS

TITLE-ABS-KEY ( ( perimenopause* OR "menopausal transition" OR menopause* OR "menopausal women" OR "midlife women" ) AND ( "complementary therapies" OR "alternative medicine" OR "complementary and alternative medicine" OR CAM OR yoga OR acupuncture OR mindfulness OR meditation OR aromatherapy OR "herbal medicine" OR supplement* OR exercise OR "behavioral therapy" ) AND ( sleep OR "sleep disorder*" OR insomnia OR "vasomotor symptoms" OR "hot flashes" OR mood OR depression OR anxiety OR "quality of life" OR fatigue ) ) AND PUBYEAR > 2013 AND PUBYEAR < 2025 AND ( EXCLUDE ( EXACTKEYWORD , "Systematic Review" ) OR EXCLUDE ( EXACTKEYWORD , "Review" ) OR EXCLUDE ( EXACTKEYWORD , "Nonhuman" ) OR EXCLUDE ( EXACTKEYWORD , "Meta Analysis" ) OR EXCLUDE ( EXACTKEYWORD , "Cohort Analysis" ) ) AND ( EXCLUDE ( PUBSTAGE , "aip" ) ) AND ( EXCLUDE ( DOCTYPE , "cr" ) OR EXCLUDE ( DOCTYPE , "bk" ) OR EXCLUDE ( DOCTYPE , "er" ) OR EXCLUDE ( DOCTYPE , "sh" ) OR EXCLUDE ( DOCTYPE , "cp" ) OR EXCLUDE ( DOCTYPE , "le" ) OR EXCLUDE ( DOCTYPE , "ed" ) OR EXCLUDE ( DOCTYPE , "no" ) OR EXCLUDE ( DOCTYPE , "re" ) OR EXCLUDE ( DOCTYPE , "ch" ) ) 

COCHRANE

(perimenopause OR perimenopausal OR "menopausal transition" OR menopause OR "menopausal women" OR "midlife women") AND ("complementary therapies" OR "alternative medicine" OR "complementary and alternative medicine" OR CAM OR yoga OR acupuncture OR mindfulness OR meditation OR aromatherapy OR "herbal medicine" OR supplements OR exercise OR "behavioral therapy") AND (sleep OR "sleep disorder" OR insomnia OR "vasomotor symptoms" OR "hot flashes" OR mood OR depression OR anxiety OR "quality of life" OR fatigue)

WOS - 753

TS=( ("perimenopause" OR "perimenopausal" OR "menopausal transition" OR "menopause" OR "menopausal women" OR "midlife women") AND ("complementary therapies" OR "alternative medicine" OR "complementary and alternative medicine" OR CAM OR yoga OR acupuncture OR mindfulness OR meditation OR aromatherapy OR "herbal medicine" OR supplements OR exercise OR "behavioral therapy") AND ("sleep" OR "sleep disorder" OR insomnia OR "vasomotor symptoms" OR "hot flashes" OR mood OR depression OR anxiety OR "quality of life" OR fatigue) )
